# Enhancing first-attempt success in radial artery cannulation: a PDCA-driven approach for anesthesiology residency training

**DOI:** 10.3389/fsurg.2025.1564760

**Published:** 2025-03-11

**Authors:** Meng Zhang, Shuchuan Zhao, Fangjing Bai, Shanshan Yu, Huixian Zhou, Siyuan Song, Guangmin Xu

**Affiliations:** ^1^ Department of Anesthesiology, Sichuan Provincial People’s Hospital, School of Medicine, University of Electronic Science and Technology of China, Chengdu, China; ^2^ Department of Anesthesiology, Bazhou District People's Hospital, Sichuan, China; ^3^ Internal Medicine Department, Montefiore Medical Center Wakefield Campus, New York, NY, United States; ^4^ Department of Neuroscience, Baylor College of Medicine, Houston, TX, United States

**Keywords:** Plan-Do-Check-Act, teaching method, standardized training, radial artery cannulation, anesthesiology

## Abstract

**Objective:**

This study aimed to evaluate the effectiveness of the Plan-Do-Check-Act (PDCA) cycle in improving the first-attempt success rate of radial artery cannulation among anesthesiology residents undergoing standardized training.

**Methods:**

Eighty-six residents from Sichuan Provincial People's Hospital, comprising 70 anesthesiology and 16 non-anesthesiology residents, were randomly divided into a control group and a PDCA group, each with 43 participants. Key outcomes assessed included first-attempt success rate, procedure duration, ultrasound utilization, preparation errors, and complication rates.

**Results:**

In anesthesiology residents, the PDCA group achieved a significantly higher first-attempt success rate (94%, 31/33) compared to the control group (43%, 16/37; *P* < 0.001). Among non-anesthesiology residents, the PDCA group also outperformed the control group, with success rates of 80% (8/10) vs. 33% (2/6; *P* = 0.048). Procedure duration was notably shorter in the PDCA group for both anesthesiology residents (median: 0.80 min, IQR: 0.50–2.30) and non-anesthesiology residents (median: 1.50 min, IQR: 0.70–3.00), compared to the control group (4.10 min, IQR: 3.10–5.90, and 3.70 min, IQR: 2.50–5.00; *P* < 0.001 and *P* = 0.026, respectively). Additionally, ultrasound usage was higher in the PDCA group, and assessment scores showed improvement, though the latter did not reach statistical significance.

**Conclusion:**

The PDCA cycle significantly enhances the first-attempt success rate and efficiency of radial artery cannulation while promoting greater adoption of ultrasound. These findings highlight its value in advancing standardized training for anesthesiology residents.

## Introduction

Radial artery puncture, though a commonly performed and relatively low-risk procedure in anesthesia, carries potential complications. These include arterial spasm, hematoma formation at the puncture site, secondary infections, induced heart failure, arterial occlusion, pseudoaneurysm, distal ischemia, and neurovascular damage (1, 2). Therefore, enhancing radial artery catheterization techniques is essential for increasing first-attempt success rates and minimizing associated risks.

With advancements in medical technology and rising expectations for healthcare services, there is a greater emphasis on improving the professional competence and service quality of medical practitioners. Standardized residency training plays a pivotal role in preparing the future healthcare workforce, as its quality directly influences the overall standard of medical care. Anesthesia, a specialty characterized by complex and high-stakes clinical responsibilities, highlights the critical need for comprehensive and effective training. By providing systematic instruction and assessment, standardized residency programs enable trainees to acquire professional expertise more efficiently, enhance their clinical decision-making and adaptability, and ultimately deliver superior medical care while prioritizing patient safety (3–5).

The Plan-Do-Check-Act (PDCA) cycle is a continuous improvement framework that effectively supports the quality management of standardized residency training. This model facilitates structured planning, implementation, evaluation, and refinement of training processes. The cycle begins with defining clear objectives, developing detailed plans and assessment criteria, and aligning the curriculum with real-world clinical demands. During the “Do” phase, theoretical lessons, clinical mentorship, and hands-on skills training are implemented to ensure residents gain the necessary knowledge and experience. In the “Check” phase, regular evaluations and feedback surveys help identify areas requiring improvement. The “Act” phase focuses on addressing identified gaps by implementing targeted measures and incorporating lessons learned to inform subsequent iterations of the PDCA cycle (6–8).

## Methods

### PDCA cycle implementation

**Figure 1:**
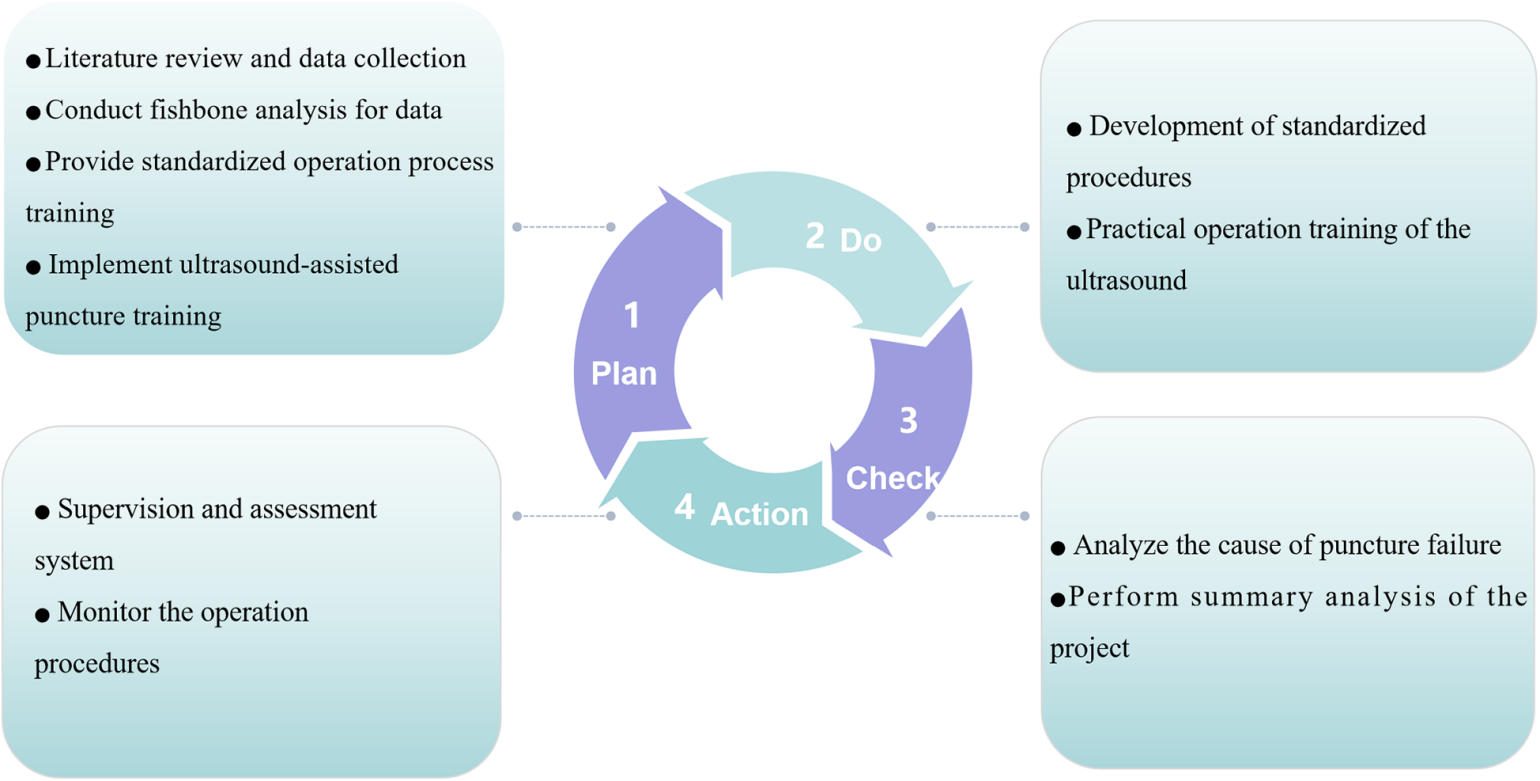
PDCA cycle utilized for developing a training plan and optimizing quality control.

**Figure 2:**
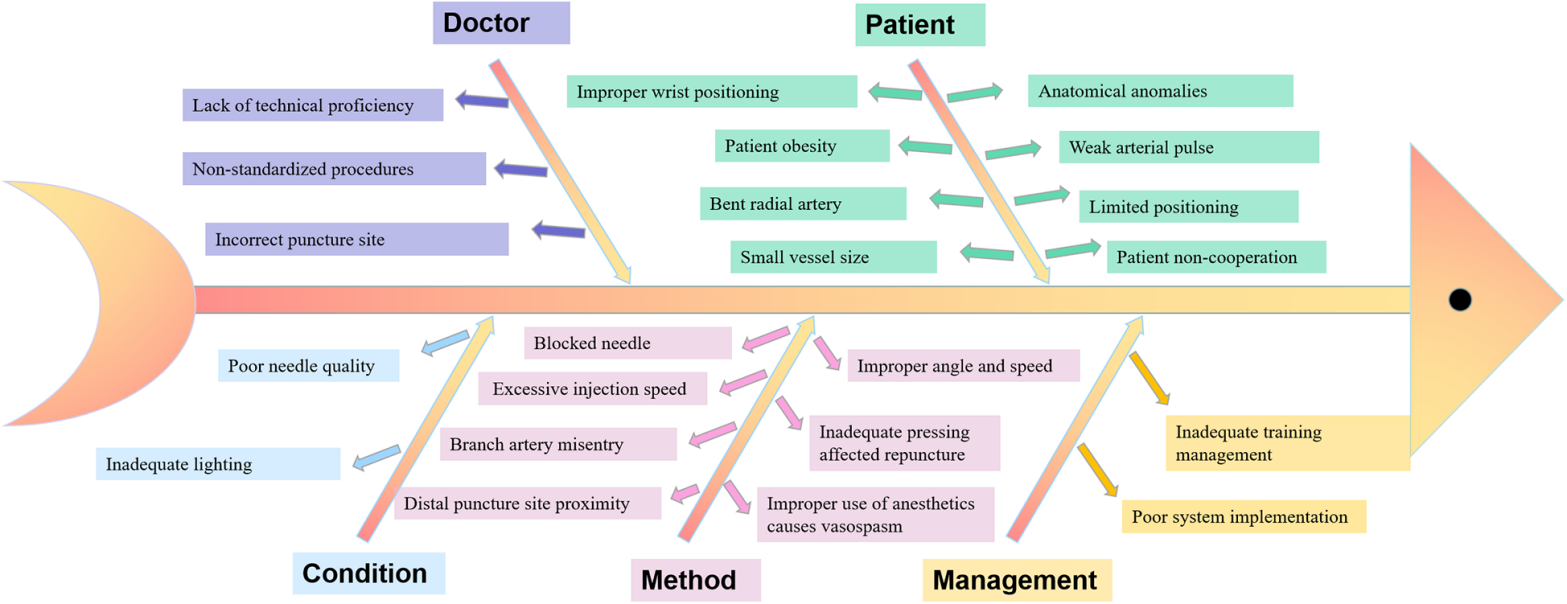
Fishbone diagram summarizing factors influencing the success rate of radial artery cannulation.

The PDCA cycle was utilized to design a structured training plan and implement quality control measures (Figure 1). A fishbone diagram was employed to systematically analyze factors influencing the success rate of radial artery cannulation (Figure 2).

### Study participants and group assignment

Eighty-six residents from Sichuan Provincial People's Hospital participated, including 70 anesthesiology residents and 16 from non-anesthesia specialties. Participants were randomly allocated into two groups: the control group and the PDCA group, each comprising 43 residents. The control group included 37 anesthesiology residents and 6 from non-anesthesia specialties, while the PDCA group consisted of 33 anesthesiology residents and 10 from non-anesthesia specialties. Performance was evaluated based on predefined procedural and operational criteria, without the use of formal questionnaires or assessments. The study adhered to all relevant guidelines and regulations and was approved by the Ethics Committee of the Affiliated Hospital of the University of Electronic Science and Technology, Sichuan Provincial People's Hospital (Approval notice: 2024-475). No human specimens were involved, and the study did not fall under the scope of ethical review for biomedical research involving human subjects. Informed consent was obtained from all participants or their legal guardians.

### Teaching methods including PDCA implementation

Control Group: Traditional teaching methods were employed. Residents first reviewed national standardized training materials, covering the theoretical principles of radial artery puncture and catheterization. These included radial artery anatomy, the purpose and measurement of Allen's test, procedural steps for puncture and catheterization, and strategies to prevent and manage complications (9). On training day, the instructor conducted one-on-one demonstrations, explained critical procedural steps, and addressed residents’ questions during the session (6).

PDCA Group: Plan: A review of relevant literature was conducted to refine the training program and define clear objectives (10, 11). A fishbone diagram was used to identify factors impacting success rates, and specific management goals were established by the PDCA project team. Do: Standardized operating procedures were developed based on national residency training guidelines. Training materials, including slides, videos, and bedside instruction, were distributed. Monthly sessions were conducted to ensure comprehensive coverage. Residents began as first assistants for at least five procedures before qualifying as primary operators. Ultrasound was mandated for vascular assessment and puncture site localization prior to the procedure. Check: Supervisors monitored compliance with standardized procedures and maintained detailed records of operators and procedural circumstances. Act: Factors influencing procedural success were analyzed to set goals for subsequent training phases. Identified knowledge gaps and errors were addressed in the next cycle to refine the program and enhance performance.

### Assessment method

The performance of 86 anesthesiology residents at Sichuan Provincial People's Hospital undergoing standardized training from January to May 2024 was evaluated in radial artery puncture and catheter placement. The assessment criteria included the following: (1) Preparation Quality: The number of preoperative material items missed during preparation. (2) Procedural Steps: Evaluation of key procedural components, including preparation of the pressure kit and arterial pressure zeroing, disinfection and administration of local anesthesia, puncture angle and positioning, catheter insertion and fixation, and maintenance of aseptic techniques. Each category was scored on a scale of 0–20. (3) First-Attempt Success Rate: The proportion of successful first attempts. If the first two attempts were unsuccessful, the supervising physician completed the procedure. (4) Procedure Duration: Time required to complete the procedure. (5) Ultrasound Utilization: The rate of ultrasound use during the procedure. (6) Complication Rates: Occurrence of complications during or after the procedure.

### Observation indicators

The performance of the control and PDCA groups was evaluated based on the following key indicators: (1) First-Attempt Success Rate: The proportion of successful radial artery cannulations on the first attempt. (2) Procedure Duration: The time required to complete the radial artery puncture and catheter placement. (3) Ultrasound Utilization Rate: The frequency of ultrasound use during the procedure. (4) Complication Incidence: The occurrence of complications such as local hematoma, ecchymosis, infection, pseudoaneurysm, thrombosis, embolism, and other related issues. (5) Preparation Accuracy: The number of missed items during the preparation phase. (6) Operation Standardization Score (assessment score): A 100-point evaluation of procedural adherence, including preparation of the pressure kit and arterial pressure zeroing, disinfection and administration of local anesthesia, puncture angle and positioning, catheter insertion and fixation, and maintenance of aseptic techniques.

### Statistical analysis

Data analysis was conducted using SPSS 22.0 software. Variables following a normal distribution were presented as mean ± standard deviation, while those not conforming to normality were reported as medians with interquartile ranges. Categorical data were expressed as frequencies and percentages. The Shapiro–Wilk test was used to evaluate normality, and Levene's test assessed variance homogeneity. For comparisons between groups, *t*-tests were applied to normally distributed data with homogeneous variance. Non-normally distributed data or data with unequal variances were analyzed using nonparametric methods, such as the Mann–Whitney *U*-test. Categorical variables were compared using the Chi-square (*χ*^2^) test, and Fisher's exact test was employed when expected frequencies were low. Statistical significance was set at α = 0.05 for all analyses.

## Results

### Baseline characteristics of participants

A total of 70 anesthesiology residents and 16 non-anesthesiology residents undergoing standardized training at Sichuan Provincial People's Hospital were randomly allocated to the control or PDCA group, with 43 participants in each group. Baseline characteristics, including age, gender, and education level, were comparable between the two groups, with no statistically significant differences (Table 1).

**Table 1:** Comparison of baseline characteristics between control and PDCA groups.

Characteristics	PDCA group	Control group	*χ*²/t	*P* value
Age (years, x¯ ± s)	25.3 ± 0.67	25.2 ± 0.77	0.48	0.63
Sex (male, female)	17, 26	19, 24	0.05	0.82
Education (undergraduate, postgraduate)	29, 14	30, 13	0.21	0.65
Anesthesiology residents (*n*)	33	37	0.74	0.39
Non-anesthesiology residents (*n*)	10	6	1.14	0.29

### Operational assessment results

The PDCA group achieved significantly higher first-attempt success rates for radial artery cannulation. Among anesthesiology residents, the success rate was 94% (31/33) in the PDCA group compared to 43% (16/37) in the control group (*P* < 0.001). Similarly, non-anesthesiology residents in the PDCA group demonstrated a success rate of 80% (8/10) compared to 33% (2/6) in the control group (*P* = 0.048).

The duration of radial artery puncture and catheterization was notably shorter in the PDCA group for both anesthesiology and non-anesthesiology residents. Median times were 0.80 min (IQR: 0.50–2.30) and 1.50 min (IQR: 0.70–3.00), respectively, compared to 4.10 min (IQR: 3.10–5.90) and 3.70 min (IQR: 2.50–5.00) in the control group (*P* < 0.001 and *P* = 0.026, respectively).

Additionally, the ultrasound utilization rate was significantly higher in the PDCA group compared to the control group. Among anesthesiology residents, 19 out of 33 (58%) in the PDCA group used ultrasound, whereas only 5 out of 37 (14%) in the control group did so (*χ*^2^ = 12.58, *P* < 0.001). Among non-anesthesiology residents, ultrasound usage was also higher in the PDCA group (3 out of 10, 30%) compared to the control group (1 out of 6, 17%), although this difference did not reach statistical significance (*χ*^2^ = 0.55, *P* = 0.46). While the assessment scores were slightly higher in the PDCA group (87.4 ± 2.5 for anesthesiology residents, 85.8 ± 3.4 for non-anesthesiology residents) compared to the control group (86.5 ± 3.3 and 85.2 ± 4.0, respectively), the differences were not statistically significant (*P* = 0.23 and *P* = 0.71, respectively). In terms of preparation accuracy, no missing items were recorded in either group, indicating that all residents demonstrated complete adherence to the required preparation standards (Number of items missing = 0.0 in both groups). Complication rates remained low in both groups, with no significant differences observed (0% in the PDCA group vs. 3% in the control group for anesthesiology residents, *P* = 0.36; 0% vs. 17% for non-anesthesiology residents, *P* = 0.23) (Table 2).

**Table 2:** Operational assessment of PDCA and control groups.

Assessment	PDCA group	Control group	χ²/t	*P* value
Number of first-attempt puncture success [cases, *n* (%)]				
Anesthesiology residents	31 (94%)	16 (43%)	16.22	<0.001**
Non-anesthesiology residents	8 (80%)	2 (33%)	4.00	0.045*
Duration of catheterization [min, M (Q25, Q75)]				
Anesthesiology residents	0.80 (0.50, 2.30)	4.10 (3.10, 5.90)	5.81	<0.001**
Non-anesthesiology residents	1.50 (0.70, 3.00)	3.70 (2.50, 5.00)	2.45	0.026*
Preparation accuracy Number of items missing (x¯ ± s)				
Anesthesiology residents	0.0	0.0	–	–
Non-anesthesiology residents	0.0	0.0	–	–
Ultrasound utilization rate [cases, *n* (%)]				
Anesthesiology residents	19 (58%)	5 (14%)	12.58	<0.001**
Non-anesthesiology residents	3 (30%)	1 (17%)	0.55	0.46
Assessment score (x¯ ± s)				
Anesthesiology residents	87.4 ± 2.5	86.5 ± 3.3	1.22	0.23
Non-anesthesiology residents	85.8 ± 3.4	85.2 ± 4.0	0.38	0.71
Incidence of complications [cases, *n* (%)]				
Anesthesiology residents	0 (0%)	1 (3%)	0.85	0.36
Non-anesthesiology residents	0 (0%)	1 (17%)	1.43	0.23

* *P* < 0.05.

** *P* < 0.01.

## Discussion

Radial artery puncture and cannulation are critical skills in anesthesiology, essential for accurate monitoring and patient care (12). However, improper technique or errors can lead to complications such as hematoma, infection, arterial injury (e.g., spasm or entrapment), thrombosis, and compromised blood supply to the hand. These complications not only endanger patient safety but also hinder the efficiency of clinical anesthesia practice, underscoring the need for comprehensive and effective training (13).

Anesthesiology residents often face challenges stemming from limited practical experience and knowledge. The complex conditions of patients in clinical settings further increase the demands on trainees (14). Traditional teaching methods, which heavily rely on clinical internships, are often hindered by the fast-paced clinical environment, high staff turnover, and the psychological stress experienced by patients. Additionally, the inconsistency in teaching methods across instructors can leave residents confused, anxious, and struggling to effectively learn and apply critical skills, leading to prolonged internships (15, 16).

To address these limitations, the PDCA cycle was adopted to train residents in radial artery puncture and catheterization at teaching hospitals. The benefits of this method include: (1) Structured Knowledge Transmission: The PDCA approach enables the delivery of a complete and coherent standard operating process within a relatively short timeframe, helping residents form accurate foundational knowledge and skills. (2) Reduced Anxiety: Monthly slide presentations, videos, and bedside lectures provide a consistent and focused learning environment, reducing nervousness and enhancing retention. (3) Practical Relevance: Training materials, including slides, videos, and bedside teaching, use equipment and supplies consistent with clinical practice, such as heparin formulations, sterile packs, and monitors. This alignment helps reduce preparation errors, as evidenced by significantly fewer missed preparation items in the PDCA group compared to the control group. (4) Reinforcement and Proficiency: Saved training materials allow residents to review and practice independently. The PDCA cycle's structured quality management contributed to significantly higher first-attempt success rates and shorter procedure durations in the PDCA group compared to the control group. The integration of bedside teaching with ultrasound further strengthened the training (17–19). Ultrasound, as a visual and interactive tool, enhances understanding of radial artery anatomy, including depth, diameter, and location. Rotational exercises with student volunteers provided hands-on practice, helping residents refine aseptic techniques and needle insertion skills. This approach increased engagement, reduced first-time procedural anxiety, and minimized the risk of patient-related complications.

The PDCA cycle's goal-oriented approach emphasizes “accuracy, stability, and speed” as fundamental principles for residents. Adequate preparation and thorough understanding of vascular anatomy are prioritized to avoid unnecessary punctures. While procedural speed is encouraged, it must not compromise precision and stability.

## Conclusion

The study highlights the PDCA cycle as an effective and versatile approach for teaching radial artery puncture and cannulation. By emphasizing continuous planning, implementation, evaluation, and improvement, the PDCA cycle optimizes the training process and enhances residents’ technical proficiency. Its adaptability makes it well-suited to various clinical teaching scenarios, supporting broader improvements in medical education.

### Limitations

This study was conducted at a single center with a relatively small sample size, which limits the generalizability of the findings. Future multi-center studies with larger sample sizes are needed to validate these results and further explore the potential of the PDCA cycle in clinical education.

## Data Availability

The original contributions presented in the study are included in the article/Supplementary Material, further inquiries can be directed to the corresponding authors.
